# Differently Expression Analysis and Function Prediction of Long Non-coding RNAs in Duck Embryo Fibroblast Cells Infected by Duck Tembusu Virus

**DOI:** 10.3389/fimmu.2020.01729

**Published:** 2020-08-04

**Authors:** Yun Lin, Jing Yang, Dalin He, Xudong Li, Jing Li, Yi Tang, Youxiang Diao

**Affiliations:** ^1^College of Animal Science and Technology, Shandong Agricultural University, Tai'an, China; ^2^Shandong Provincial Key Laboratory of Animal Biotechnology and Disease Control and Prevention, Shandong Agricultural University, Tai'an, China; ^3^Shandong Provincial Engineering Technology Research Center of Animal Disease Control and Prevention, Shandong Agricultural University, Tai'an, China

**Keywords:** DTMUV infection, DEFs, lncRNA expression, gene regulation, RNA-Seq

## Abstract

Duck Tembusu virus (DTMUV), the causative agent of egg-drop syndrome, has caused substantial economic losses to duck industry. DTMUV infection leads to profound changes of host cells, including transcriptome and proteome. However, the lncRNA expression profile and the biological function of lncRNA have not been revealed. Therefore, DTMUV was used to inoculate duck embryo fibroblast cells (DEFs) for high-throughput RNA-sequencing (RNA-Seq). The results showed that 34 and 339 differently expressed lncRNAs were, respectively, identified at 12 and 24 h post-infection (hpi). To analyze their biological functions, target genes in cis were searched and the regulatory network was formed. Kyoto Encyclopedia of Genes and Genomes (KEGG) enrichment analysis revealed that the target genes were strongly associated with immune system, signaling molecular and interaction, endocrine system, and signal transduction. The differently expressed lncRNAs were selected and verified by quantitative real-time polymerase chain reaction (RT-qPCR). Our study, for the first time, analyzed a comprehensive lncRNA expression profile in DEFs following DTMUV infection. The analysis provided a view on the important roles of lncRNAs in gene regulation and DTMUV infection.

## Introduction

In 2010, a novel disease characterized by a significant decline in egg-drop production broke out in many duck farms across Chain (1). The disease, diagnosed as duck hemorrhagic ovaritis, was finally proven to be caused by DTMUV (2–4). DTMUV, similar to other flaviviruses, is a single-stranded, positive-sense RNA virus with an approximately 11 kb genome. It can infect not only ducks but also geese (5), chickens (6), sparrows (5), pigeons (7), and mice (6). Interestingly, a wide spectrum of mammalian cells, including A549, BHK21, Hela, Vero, and SH-SY5Y, exhibit obvious cytopathic effects (CPEs) after DTMUV infection (8). Our previous study showed that CPEs were appeared on HEK293 when the cells were infected with DTMUV, which implies that the virus with the possibility to infect human can potentially threaten human health (9).

LncRNAs, more than 200 nucleotides in length, were recognized as pseudo-transcriptions due to the lack of protein-coding capacity (10). In recent years, they were implicated in complex biological processes through diverse mechanisms such as in gene regulation by titration of transcription factors, splicing alteration, sponging of microRNAs, and recruitment of chromatin modifying enzymes (11–14). Besides, they could function in cis to regulate expression of neighboring gene and in trans to impact gene expression across chromosomes (15). And emerging evidence uncovered that lncRNAs, induced by various viruses, were considered to regulate host innate immune response (16).

RNA-Seq, promising simultaneous transcript discovery and abundance estimation, is more powerful for revelation of transcriptome complexity and for identification of non-coding RNAs, new transcription units, and alternative splicing (17, 18). Recently, it has been widely used to reveal the expression levels of RNA transcripts in specific tissues or cells in different physiological states and cellular environments (19). Unquestionable, it provides important insights into the interaction mechanism between pathogen and host (20–22).

Up to now, extensive and in-depth researches about pathogen (5, 23, 24), pathogenicity (6, 25, 26), epidemiology (5, 27), rapid diagnosis (28–30), and vaccine (31) were carried out to prevent and control DTMUV infection. Recently, we revealed the expression profile and biological function of mRNAs in DTMUV-infected DEFs (8). However, the expression and function of lncRNAs in response to DTMUV infection remain superficial. Therefore, we analyzed a comprehensive lncRNA expression profile in DEFs following DTMUV infection. Besides, the cis target genes of differentially expressed lncRNAs were identified and then used for Gene Ontology (GO) and KEGG enrichment analyses to elucidate their biological processes and associated pathways. Our findings contribute to further understanding of the regulatory mechanism of lncRNAs. In addition, the analysis provides a new perspective for DTMUV-host interaction.

## Materials and Methods

### Cell Culture and Virus Infection

Freshly isolated DEFs were obtained from 10-day-old specific pathogen free (SPF) duck embryos (purchased from Harbin Veterinary Research Institute, Harbin, China). DEFs were cultured in Dulbecco's modified Eagle's medium (DMEM/F-121:1) (01-172-1ACS, BI, Kibbutz, Beit Haemek, Israel) supplemented with 10% fetal bovine serum (FBS) (04-001-1ACS, BI, Kibbutz, Beit Haemek, Israel), 100 μ/mL penicillin, and 100 μg/mL streptomycin (P1400, Solarbio Science & Technology Co., Ltd., Beijing, China) at 37°C with 5% CO_2_. When reaching 80–90% confluency, DEFs were mock-infected or infected with DTMUV (SDSM strain, GenBank Accession No. KC333867.1, which was obtained from our laboratory, the Poultry Disease Lab of Shandong Agricultural University) at a multiplicity of infection (MOI) of 3. After viral adsorption for 1.5 h at 37°C with 5% CO_2_, the inoculum was replaced with maintenance medium (DMEM/F-12 with 2% FBS) for further maintaining. The virus replication was detected at 12, 24, and 48 hpi. Each sample had three biological replicates.

This study was approved by the Committee on the Ethics of Animal of Shandong (permit number 20,156,681). All subjects gave informed consent for their participation in the study.

### RNA Isolation and RNA-Seq

The total RNA was extracted using TRIzol reagent (Vazyme Biotech Company, China) according to the manufacturer's instructions. The concentration of total RNA was determined using a Nanodrop instrument (Thermo Fisher Scientific). RNA quality was assessed by the detection of the A260/A280 ratio, with a value of 1.8–2.0 indicating high quality. Ribo-zero-magnetic-kit (Epicenter, USA) was used to remove ribosomal RNA from the samples. RNA libraries were prepared using TruSeq RNA LT Sample Prep Kit v2 (Illumina, San Diego, CA, USA). Library sequencing was performed on an Illumina Hiseq3000 platform by the Shanghai Personal Biotechnology (Shanghai, China).

### Bioinformatic Analyses

Clean data were obtained by removing adaptors, poly-N sequences, and poor-quality using Cutadapt software (http://cutadapt.readthedocs.io/en/stable/). Quality control analysis was performed on clean data using FastQC software (http://www.bioinformatics.babraham.ac.uk/projects/fastqc). The filtered reads were then mapped to the Peking duck reference genome (duckbase.refseq.v4.fa, http://www.duckbase.org/Download) using TopHat2 software (http://tophat.cbcb.umd.edu/) (32, 33). The transcripts were assembled with the mapped reads using StringTie software (http://ccb.jhu.edu/software/stringtie/) (34).

### Coding Potential Analyses and Differential Expression Analyses

The coding ability of lncRNAs was predicted using three tools, including Coding-Non-Coding-Index (CNCI) (https://github.com/www-bioinfo-org/CNCI) (35), Coding Potential Calculator (CPC) (http://cpc.cbi.pku.edu.cn/) (36), and Pfam-scan (http://www.ebi.ac.uk/Tools/pfa/pfamscan/help/) (37). The intersecting no-coding transcripts of the three tools were designated as credible lncRNAs. The DESeq software (http://www.bioconductor.org/packages/release/bioc/html/DESeq.html) (38) was used to perform differential expression analyses. A *p* < 0.05 and |fold change| ≥ 2 were set as the threshold for significantly differential expression.

### Target Gene Prediction and Function Analyses

In order to explore the role of differently expressed lncRNAs in regulating gene expression, cis analyses were implemented to predict the target genes. The known protein-encoding genes located within a 100-kb window upstream or downstream of lncRNAs were identified as cis target genes. To assess biological function of target genes, GO enrichment analysis basing on GO database (the date of the update of the database is January 1st, 2018) was performed using the topGO software (http://www.bioconductor.org/packages/release/bioc/html/RamiGO.html) (39). In this analysis, biological function was mainly classified into molecular function, biological process, and cellular component. Only categories with a *p* < 0.05 were considered significantly enriched. In addition, the associated pathways of cis target genes were predicted by KEGG database (the date of the update of the database is January 1st, 2018). The signal pathway terms with a *p* < 0.05 were considered significantly enriched.

### RT-qPCR Analysis

The duck glyceraldehyde-3-phosphate dehydrogenase (GAPDH) gene served as the endogenous reference gene. All the primers, synthesized by TSINGKE Biological Technology (China), are listed in Table S1. RT-qPCR was carried out on a Light Cycler 480II instrument (Roche, Basel, Switzerland) using One Step TB Green PrimeScript™ RT-PCR Kit (TaKaRa, Dalian, China) according to the manufacturer's instructions, and melting curves were obtained. The relative expression levels of DTMUV and differentially expressed lncRNAs were calculated through 2^−ΔΔCt^ method (40). Each sample had three biological replicates. Statistical analyses were performed using Student's *t*-tests. We performed correlation analysis between RNA-Seq and RT-qPCR with GraphPad Prism software, Version 7.0.

## Results

### Confirmation of DTMUV Infection in DEFs

**Figure 1:**
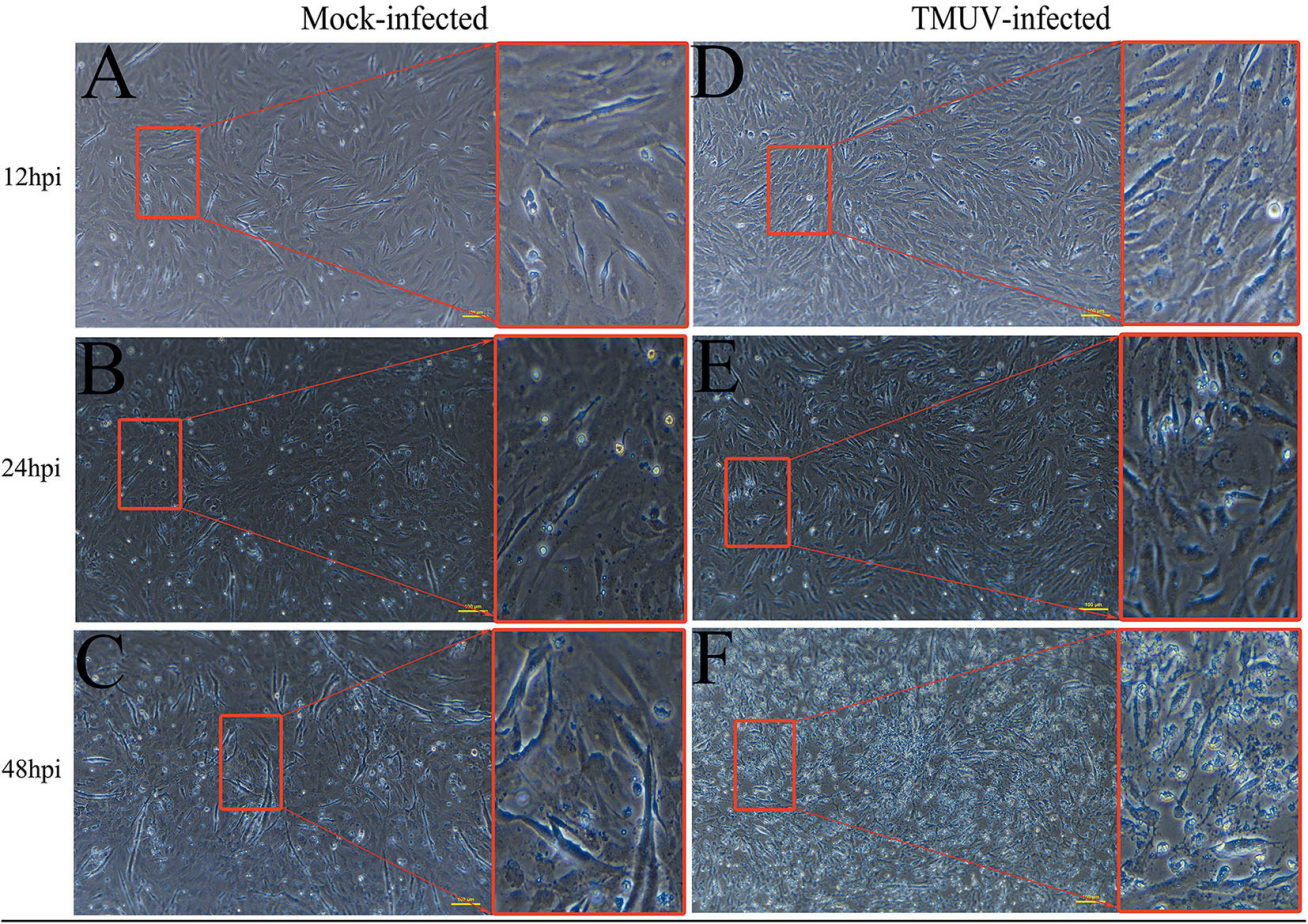
Cytopathic effects of duck embryo fibroblast cells following DTMUV infection at 12, 24, and 48 h post-infection. Each sample had three biological replicates. The yellow scale bar represents 100 μm. **(A)** The status of mock-infected DEFs at 12 hpi. **(B)** The status of mock-infected DEFs at 24 hpi. **(C)** The status of mock-infected DEFs at 48 hpi. **(D)** The status of TMUV-infected DEFs at 12 hpi. **(E)** The status of TMUV-infected DEFs at 24 hpi. **(F)** The status of TMUV-infected DEFs at 48 hpi.

**Figure 2:**
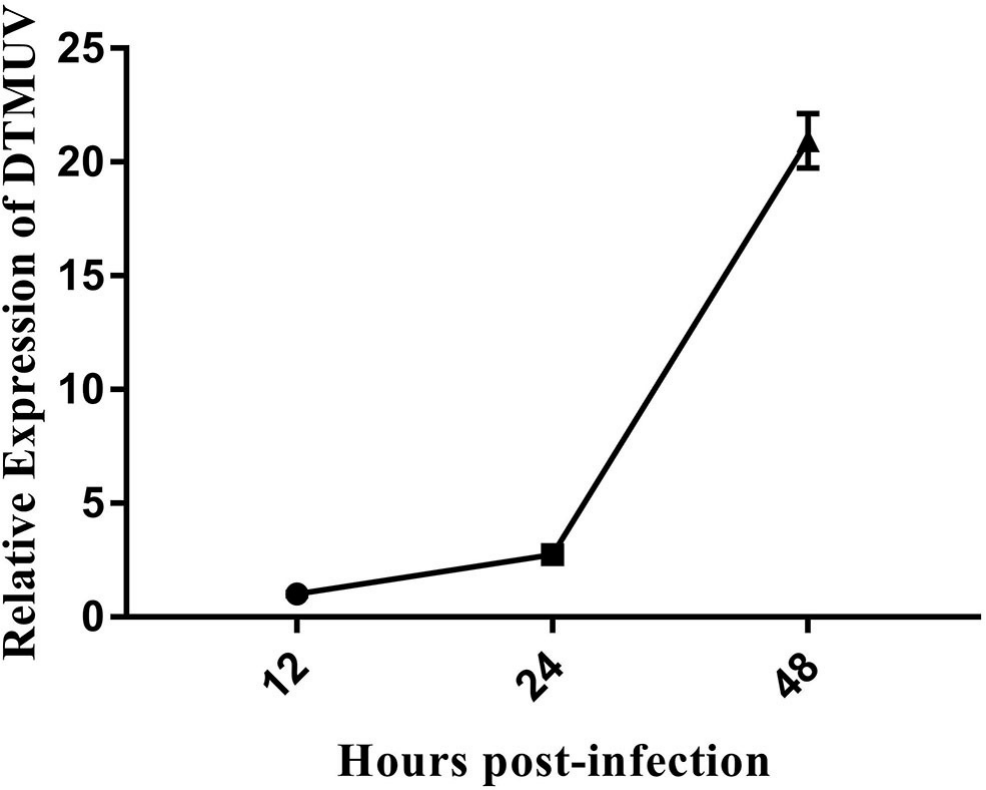
The DTMUV replication in duck embryo fibroblast cells following infection at 12, 24, and 48 h post-infection. Each sample had three biological replicates.

DEFs were infected with DTMUV at a MOI of 3. The successful infection was verified by observation of CPE and determination of virus replication monitored by RT-qPCR at 12, 24, and 48 hpi. As is showed in Figure 1, no CPE was observed in mock-infected DEFs (Figures 1A–C), and CPE on DTMUV-infected DEFs at 12 hpi was not visible (Figure 1D). However, the pathological cellular state (the cells were shrinking and rounded) could be recognized at 24 hpi as early as possible (Figure 1E), and the pathological condition was more visible (the cells were shrinking, cracking, and suspending) at 48 hpi (Figure 1F). The result of virus replication was showed in Figure 2. The viral replication gradually increased at 12, 24, and 48 hpi, indicating the development of persistent infection. The DEFs at 12 and 24 hpi were harvested for RNA-Seq.

### The Code Capacity, Length Distribution, and Density Distribution of lncRNAs

**Figure 3:**
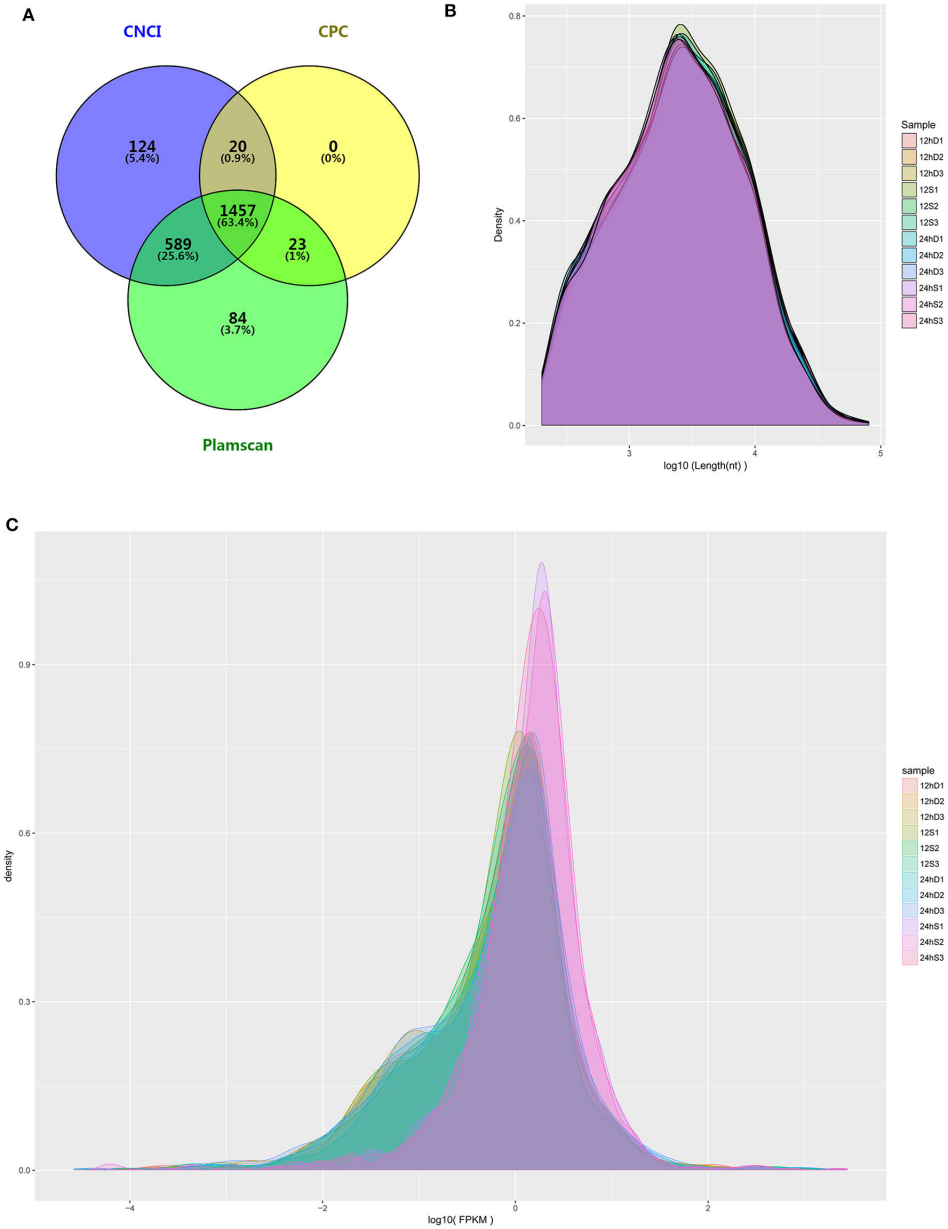
**(A)** The Venn diagrams showing the number of lncRNAs filtered by the three methods. **(B)** lncRNA length distribution. **(C)** lncRNA density distribution.

RNA-Seq was performed to determine the expression levels of lncRNAs in DEFs infected or uninfected with DTMUV. After removing adaptor and low-quality sequences, the ability of transcripts to encode protein was determined. The result showed that 1457 lncRNAs were filtrated by the three methods in common (Figure 3A). The details of the results are included in Table S2. The length and density distribution revealed that lncRNAs, 3,000–4,000 nucleotides in length and medium-expressed, occupy a dominant position in samples (Figures 3B,C).

### Differential Expression Analysis and Target Prediction

**Figure 4:**
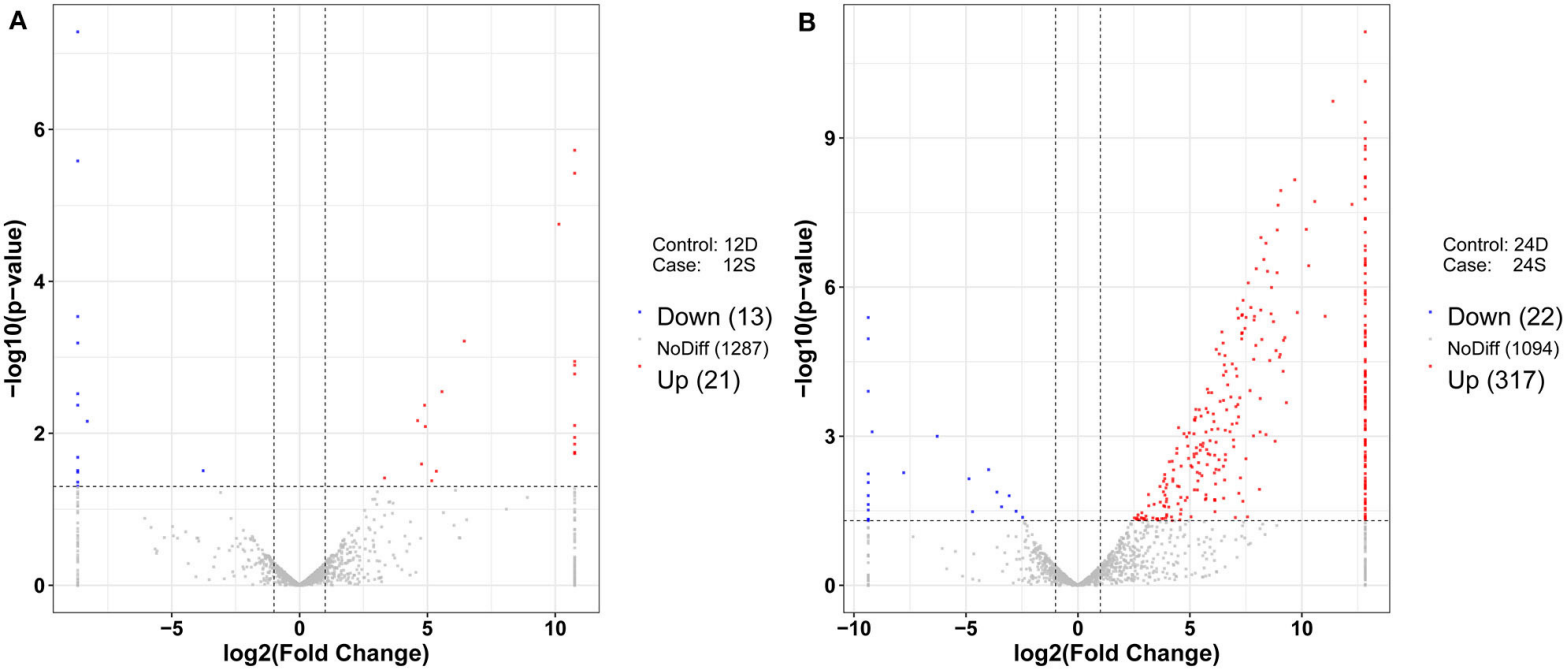
**(A)** Volcano chart of differently expressed lncRNAs at 12 hpi. **(B)** Volcano chart of differently expressed lncRNAs at 24 hpi. The x-axis shows the Log_2_ (fold change) and y-axis shows the –log_10_ (*p*-value). Red points represent the upregulated lncRNAs and green points represent the downregulated lncRNAs. The vertical line in the figure is a two-fold difference threshold, and the horizontal line is a *p* < 0.05 threshold.

**Figure 5:**
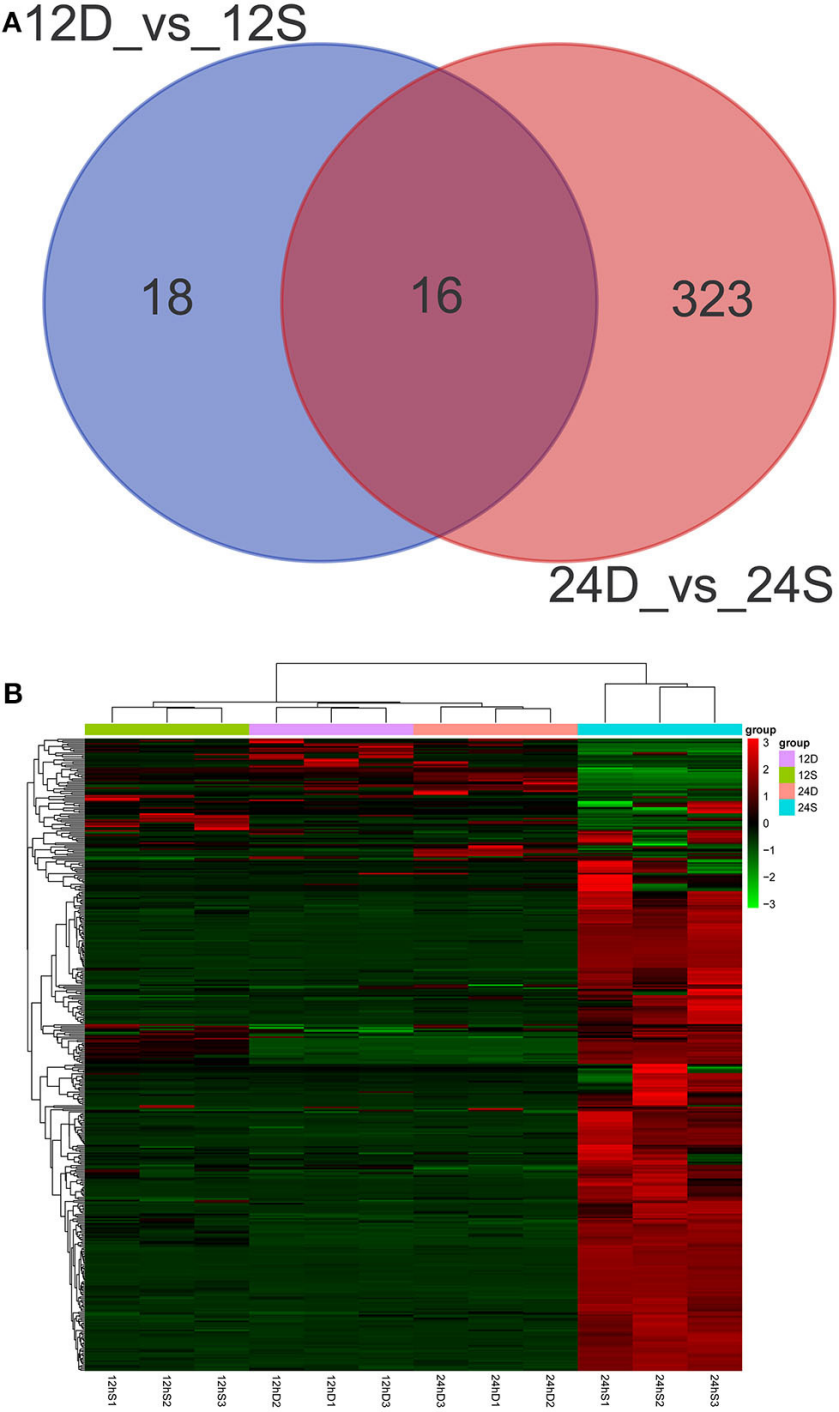
**(A)** Venn diagrams showing the numbers of differently expressed lncRNAs at 12 and 24 hpi. **(B)** The heat map showing the hierarchical clustering of altered lncRNAs. Red represents upregulation, and green represent downregulation.

**Figure 6:**
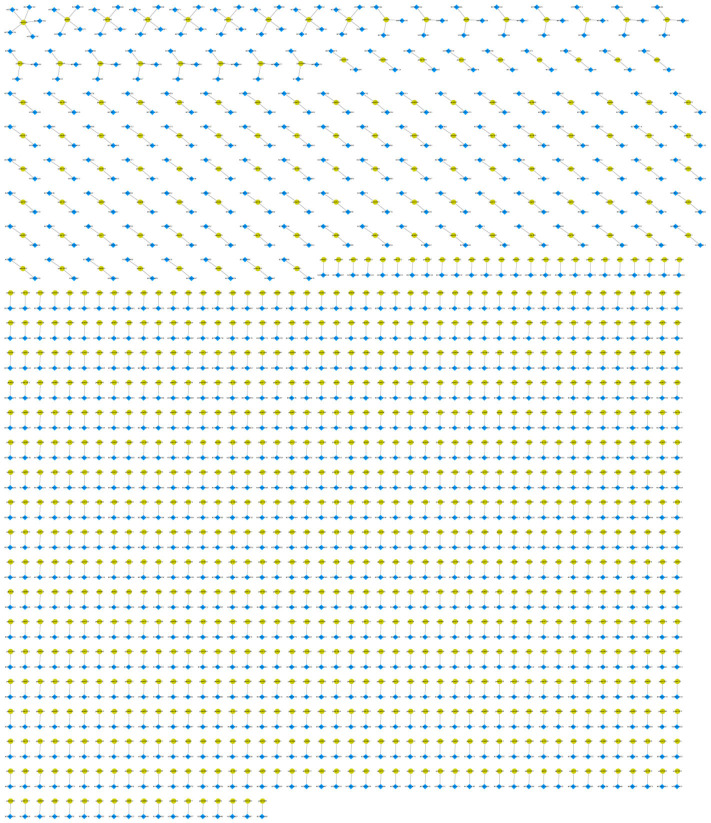
The regulatory network of differently expressed lncRNAs and cis target genes. The blue dots represent differently expressed lncRNAs, and yellow dots represent target genes.

As is shown in Figure 4, 357 differently expressed lncRNAs were identified in DTMUV-infected DEFs compared with mock-infected DEFs, among which 34 lncRNAs were identified at 12 hpi with 21 up-regulated and 13 down-regulated (Figure 4A). Respectively, 339 lncRNAs were identified at 24 hpi with 317 up-regulated and 22 down-regulated (Figure 4B). The details of the information are included in Tables S3, S4. Notably, 16 lncRNAs expressed differently at both time points (Figure 5A). The differently expressed lncRNAs were then applied to a systematic cluster analysis. Obviously, lncRNA expression levels were significantly altered at 24 hpi, while the expression profile did not differ importantly at 12 hpi (Figure 5B). To better explore the regulatory function of differently expressed lncRNAs, cis target genes have been searched through location. The regulatory network is shown in Figure 6. The details of the information are included in Table S5.

### GO and KEGG Enrichment Analysis

**Figure 7:**
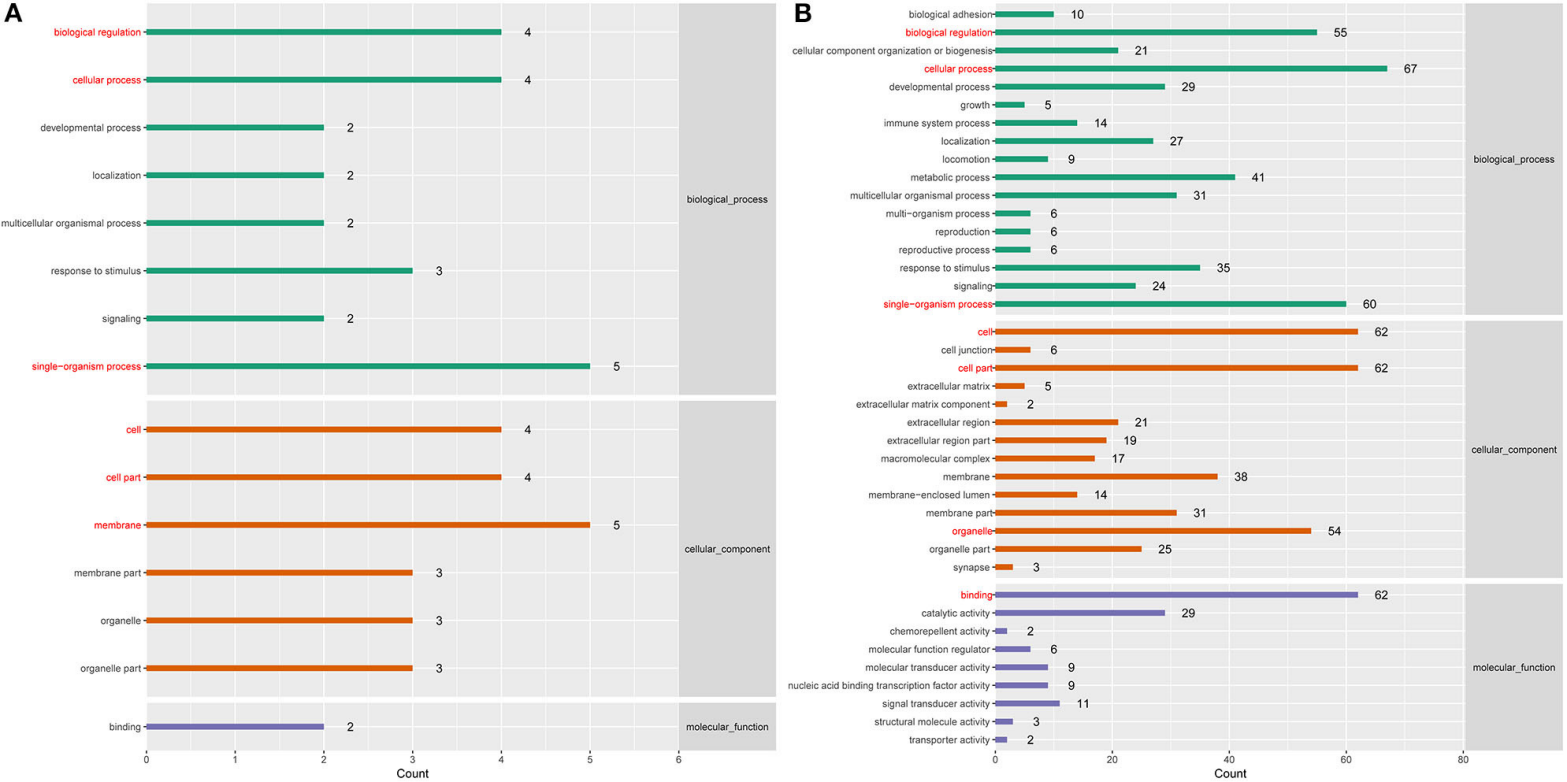
**(A)** GO enrichment analysis at 12 hpi. **(B)** GO enrichment analysis at 24 hpi. Each color represents a different biological process. The x-axis indicates the description and the y-axis indicates the –log_10_ (*p*-value).

**Figure 8:**
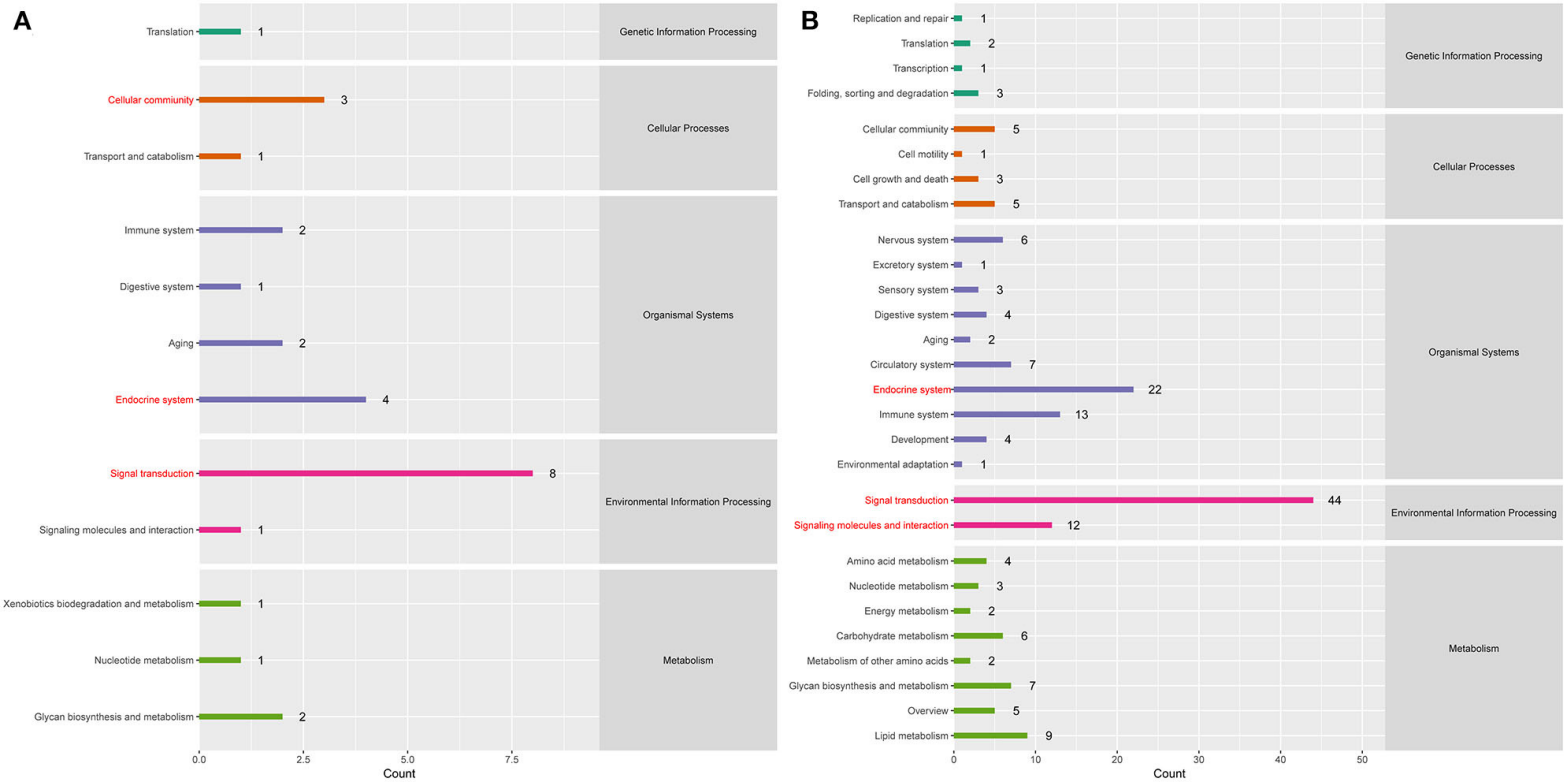
**(A)** KEGG pathway analysis at 12 hpi. **(B)** KEGG pathway analysis at 24 hpi. Each color represents a kind of signal pathway.

**Figure 9:**
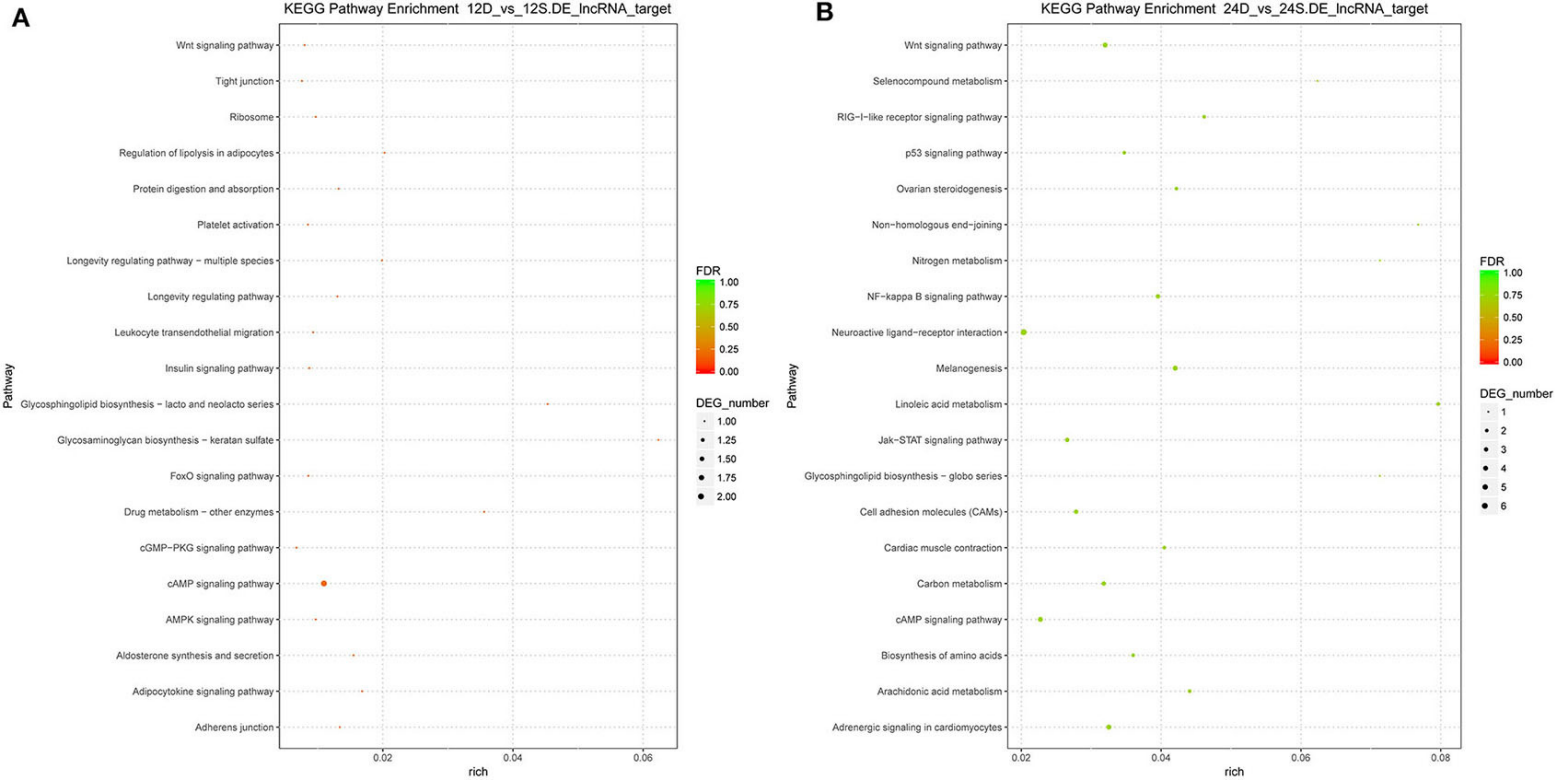
**(A)** The significantly enriched pathways at 12 hpi. **(B)** The significantly enriched pathways at 24 hpi. The x-axis and y-axis represent enrichment and pathway names, respectively. Point size represents the number of target genes.

To better understand the roles of differentially expressed lncRNAs in DTMUV-infected DEFs, GO and KEGG analyses were performed to explore the biological function. As is shown in Figure 7, the target genes were mainly related to biological regulation, cellular processes, single-organism processes, cell, cell part, and membrane at 12 hpi (Figure 7A). And organelle and binding were additionally enriched in at 24 hpi (Figure 7B). The details of the GO terms are included in Tables S6, S7. Besides, target genes were closely referred to the signal pathway categories of signal transduction, endocrine system, and cellular community at 12 hpi (Figure 8A). Additionally, immune system and signaling molecules and interaction were enriched in at 24 hpi (Figure 8B). The details of the KEGG terms are included in Tables S8, S9. The significantly enriched pathways are presented in Figure 9A at 12 hpi and Figure 9B at 24 hpi.

### Validation of Differentially Expressed lncRNAs by RT-qPCR

**Figure 10:**
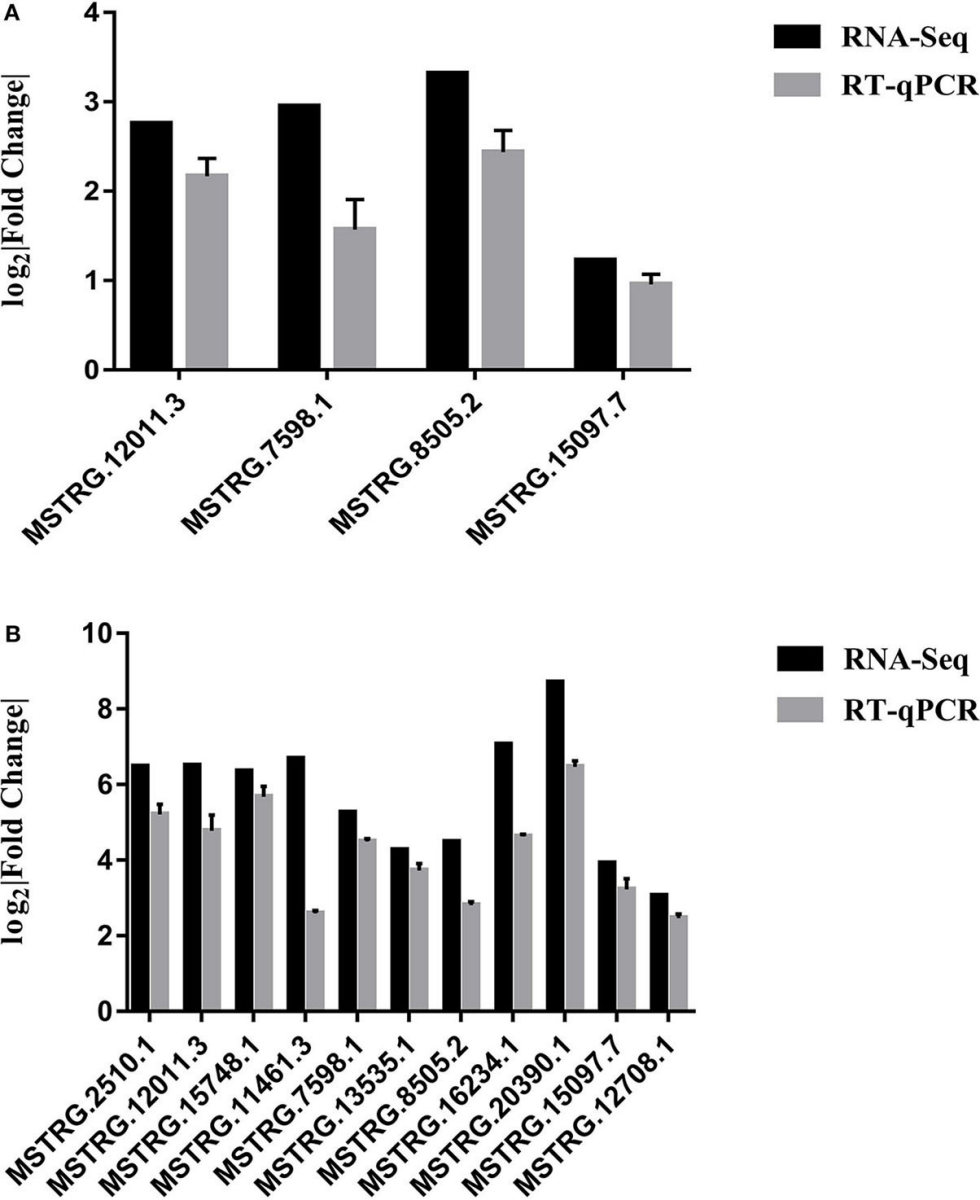
**(A)** Validation of differentially expressed lncRNAs by RT-qPCR at 12 hpi. **(B)** Validation of differentially expressed lncRNAs by RT-qPCR at 24 hpi. Each sample had three biological replicates.

**Figure 11:**
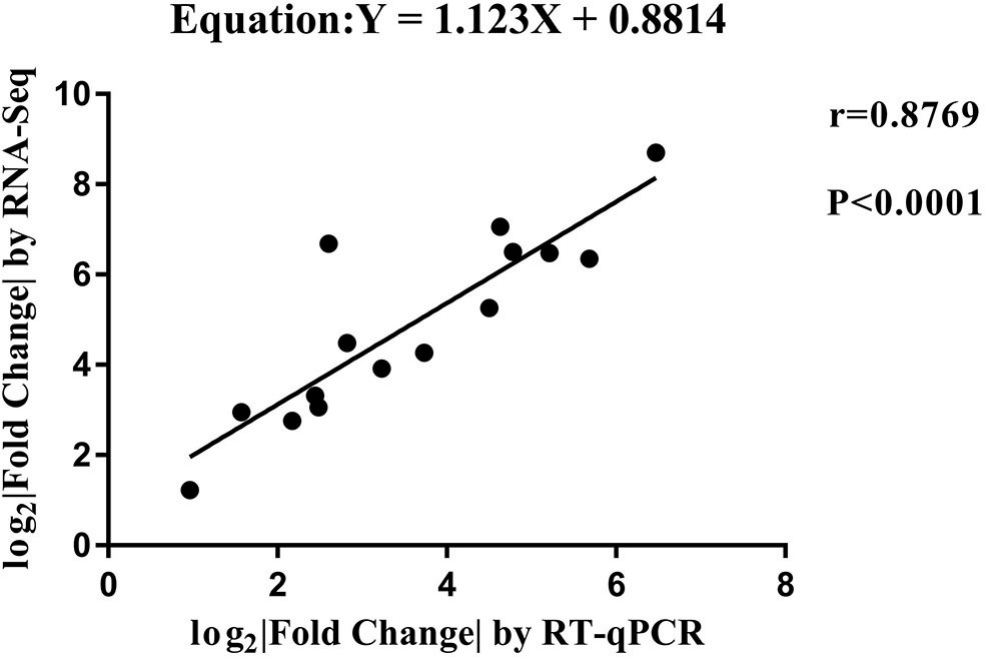
The correlation analyses of changes in lncRNA expression between RT-qPCR and RNA-Seq. The x-axis shows the Log_2_ (fold change) by RT-qPCR, and the y-axis is the Log_2_ (fold change) by RNA-Seq.

RT-qPCR was performed to further detect the expression changes of lncRNAs in RNA-Seq data. 4 and 11 lncRNAs at 12 and 24 hpi were validated by RT-qPCR. The results showed that expression changes confirmed by RT-qPCR were consistent with the RNA-Seq data (Figure 10). The details of the information are included in Table S10. Furthermore, the correlation analysis revealed that changes in lncRNA expression level were comparable between RNA-Seq and RT-qPCR, with the correlation coefficients of 0.8769 (*P* < 0.0001) (Figure 11). The results confirmed that the RNA-Seq data were relatively reliable and accurate.

## Discussion

DTMUV, an emerging member of flavivirus family, can cause acute anorexia, retarded growth, neurological dysfunction, and severe egg production drop, which results in large economic losses (41). To understand DTMUV infection deeply, the transcriptome and proteome in DTMUV-infected DEFs have been uncovered (8, 42, 43). However, little is known for lncRNA differential expression and the biological effect on gene regulation during DTMUV infection. In this study, expression changes of lncRNAs were investigated, potential regulatory network was formed, and biological function was predicted. The study provides significant insights into the deep exploration of pathogenic mechanism.

Accumulated studies demonstrated that viral infection can alter lncRNA expression profile of host (44), including infectious bronchitis virus infection of primary dendritic cells (45), H5N1 influenza viruses infection of ducks (46), and Porcine reproductive and respiratory syndrome virus infection of endometrial epithelial cells (47). In our study, the change of lncRNA expression profile was also observed in DEFs in response to DTMUV infection. To the best of our knowledge, this is the first report in which a complete lncRNA profile was provided in DTMUV-infected DEFs. The lncRNA profile showed that the expression levels of lncRNAs were specifically upregulated or downregulated in response to DTMUV infection.

Recent study showed that lncRNAs were able to cooperate with neighboring genes to perform cis regulatory function (48). For example, to control the lifespan of neutrophils, eosinophils and classical monocytes, lncRNA-Morrbid regulates the transcription of the neighboring pro-apoptotic gene, named Bcl2l11, by promoting the enrichment of the PRC2 complex at the Bcl2l11 promoter to maintain the gene in a poised state (49). Besides, LincRNA-p21, as a key modulator of gene expression in the p53 pathway, performs its cis control of p21 expression to influence the activation and chromatin state of hundreds of downstream genes (50). In our study, the cis target genes were searched and the cis regulatory network was formed.

Here, we performed GO and KEGG enrichment analysis to predict the biological function. Notably, many cis target genes were strongly associated with metabolism, such as lipid metabolism, glycan biosynthesis and metabolism, garbohydrate metabolism, and amino acid metabolism. To date, several studies have suggested that viruses regulate host metabolism to facilitate replication (51, 52). DTMUV also induced profound metabolic alterations in DEFs (8, 43). Emerging report showed that lncRNA regulates metabolic enzymes to regulate virus replication. The lncRNA ACOD1, induced in cells infected with various viruses, binds the metabolic enzyme glutamic-oxaloacetic transaminase and increases its catalytic activity to facilitate the production of metabolites that promote viral propagation (53). The findings suggested that differently expressed lncRNAs may regulate metabolism to affect the pathogenicity of DTMUV.

In addition to metabolism, the signal pathway categories of immune system were enriched in, such as RIG-I-like receptor signaling pathway. The genome-wide transcriptome analyses of DTMUV-infected macrophages revealed that the inductions of alpha interferon and beta interferon were blocked on transcription and translation levels in response to viral infection, despite the activation of major pattern recognition receptor signaling (54). Deep study showed that DTMUV non-structural protein 1 interacts with the adaptor protein mitochondrial antiviral signaling to inhibit the mitochondrial antiviral signaling pathway, resulting in the impaired induction of beta interferon (55). In addition, the non-structural protein 2B cleaves STING to inhibit interferon signaling (56). Recently, an increasing number of lncRNAs have been reported to play negative roles in innate immune response to virus infections. For example, lnc-Lsm3b, a mouse specific type I interferon-induced lncRNA, competes to bind RIG-I monomers with viral RNAs to prevent conformational changes of RIG-I and activation of its downstream signaling (57). NRAV, a down-regulated lncRNA in human epithelial cells upon influenza A virus infection, promotes virus replication by negatively regulating the transcription of multiple interferon-stimulated genes (58). Therefore, lncRNAs may participate in the invasion and infection of DTMUV through affecting the innate immunity.

## Conclusions

In summary, we were the first to perform a comprehensive analysis of lncRNA expression profile in DEFs following DTMUV infection using RNA-Seq. We screened out numerous differently expressed lncRNAs, formed cis regulatory network, and conducted biological function analysis. Our results suggested that lncRNAs may participate in DTMUV-induced pathogenesis through affecting the metabolism and innate immunity of host cells, which provides a deeper insight into the pathogenic mechanism of DTMUV. Future investigations will be required to discover specific pathogenic mechanism and to identify novel and efficient strategies for DTMUV infection.

## Data Availability Statement

The datasets generated for this study can be found in the Sequence Read Archive of the National Center for Biotechnology Information (accession number SRP150572).

## Ethics Statement

The animal study was reviewed and approved by Committee on the Ethics of Animal of Shandong (permit number 20,156,681).

## Author Contributions

YL and JY completed the experiments, analyzed the results, and drafted the work with the help of DH, XL, and JL. YT and YD provided the materials, designed the experiments, and reviewed the manuscript. All authors have read and approved the manuscript for publication.

## Conflict of Interest

The authors declare that the research was conducted in the absence of any commercial or financial relationships that could be construed as a potential conflict of interest.
